# Strategies for recruiting injection drug users for HIV prevention services in Delhi, India

**DOI:** 10.1186/1477-7517-10-16

**Published:** 2013-09-25

**Authors:** Waimar Tun, Mary Philip Sebastian, Vartika Sharma, Ira Madan, Samir Souidi, Dean Lewis, Ibou Thior, Avina Sarna

**Affiliations:** 1 Population Council, 4301 Connecticut Avenue, NW Suite 280, Washington DC 20008, USA; 2 Population Council, Zone 5A, Ground Floor, India Habitat Centre, Lodi Road, New Delhi 110003, India; 3 Sahara Centre for Residential Care and Rehabilitation, B-9/11, Chandan Singh Market, IGNOU Road, Saidulajab, New Delhi 110030, India; 4 Population Council, 1 Dag Hammarskjold Plaza, New York 10017, USA; 5 PATH, 455 Massachusetts Avenue NW, Suite 1000, Washington DC 20001, USA

**Keywords:** Injection drug user, HIV prevention, Out-reach worker, Peer-referral, Harm-reduction

## Abstract

**Background:**

We utilized multiple recruitment approaches to recruit IDUs in a longitudinal cohort study to examine HIV incidence and behavior change pre- and post-introduction of comprehensive HIV prevention services.

**Methods:**

IDUs were recruited through peer referral, targeted outreach by outreach workers (ORWs) and as walk-in clients at drop-in centers. Participants received monetary compensation for participation (USD 0.80). Participants were given recruitment coupons to recruit peers (regardless of recruitment method). For peer referral, participants received a food coupon, as secondary compensation, for each peer he/she successfully recruited. We report the profile of IDUs by recruitment method, based on the baseline behavioral survey and HIV test results. Cost per IDU recruited by recruitment method was also calculated.

**Results:**

A total of 3,818 IDUs were recruited between May 2011 and October 2011. More than half of the study participants were recruited through targeted outreach (ORW: 53.6%; peer-referral: 26.3%; walk-ins: 20.1%). Of the participants who were given recruitment coupons, 92.7% recruited no peers. Those who successfully recruited at least one peer were significantly more likely to be in a stable living accommodation compared to those who did not recruit any peers (51.1% versus 42.7%; p < 0.05). Only 45.9% of the food coupons were claimed for successful recruitment of peers. Peer-referred IDUs were more likely to be living with family or relatives (50.7% versus ORW: 40.1% and walk-in: 39.8%; p < 0.001) rather than on the street or shared housings compared to the other two recruitment modes. Walk-ins were more likely than peer-referred and ORW-referred IDUs to be HIV-positive (walk-ins: 26.1%; peer-referred: 19.1%; ORW: 19.9%; p < 0.01) and have risky injection practices (walk-ins: 62.2%; ORW: 57.0%; peer-referred: 58.6%; p < 0.05). The cost per IDU recruited through ORW referral method was the most costly at USD 16.30, followed by peer-referral at USD 8.40 and walk-in at USD 7.50.

**Conclusion:**

When recruiting a large number of IDUs, using multiple recruitment modes is ideal with regard to diversification of IDU characteristics and risk profile. Although it was the most costly, ORW recruitment was more effective than the other two methods. Lack of monetary compensation for successful recruitment of peers may have hampered peer-referral.

## Background

India is home to about 177,000 injection drug users (IDUs) with an estimated HIV prevalence of 7.1% nationally [1]. Delhi has the second highest HIV prevalence in India at 18.3% and an estimated IDU population of approximately 17,000 [2]. One of the greatest challenges in the HIV prevention efforts for IDUs is effectively reaching the population with services. IDUs are a highly hidden population given their stigmatized and illegal behavior.

The National AIDS Control Organization (NACO) provides HIV prevention services to high-risk groups, including IDUs, through targeted interventions (TI) delivered by NGOs. For IDUs, TI services include behavior change communication, provision of sterile needles and syringes, condom provision, treatment for STIs, oral substitution therapy and referrals for HIV testing and anti-retroviral treatment. NGOs implement TI work through outreach workers (ORWs) and peer educators who are often individuals with a history of injection drug use.

Despite the government’s TI efforts, significant gaps in coverage exist. A 2007 population-based study found that only 53% of IDUs were counseled on HIV prevention by an ORW, 54% reported obtaining clean needles/syringes from an intervention program in the past 12 months, and only 37% had ever tested for HIV [3]. Maximizing program coverage is essential to reducing transmission and acquisition of blood-borne infections and STIs among IDUs and their sexual and injecting partners. Given these challenges, there is a need to better understand efficient ways to reach IDUs with prevention interventions.

Outreach has been an integral part of harm reduction interventions for IDUs worldwide and has been used successfully in reaching IDUs. Programs use ORWs to access, engage and recruit IDUs for services, as IDUs may be reluctant to come into a service facility and may fear interactions with traditional service providers. ORWs are typically salaried workers and are themselves former drug users or those from the same community as the drug users so that they can gain the trust of and have credibility with their target population [4-6]. Studies have shown the effectiveness of outreach programs in reducing IDU drug use, risky injection practices, unsafe sexual practices, as well as HIV infection rates [7-9].

Peer-driven interventions (PDI) have also been used in harm reduction programs worldwide [10-13]. PDIs capitalize on the drug user’s social network, a network that likely facilitates risky injection behaviors, to promote reduction of risky behaviors. Unlike the outreach model which targets the individual IDUs, PDIs target the IDUs and their network. A feature of the PDI strategy that differentiates it from simple chain referral recruitment is the use of primary and secondary incentives to reward subjects for participating (primary incentive) and for recruiting peers into the study and intervention (secondary incentives). The secondary incentives affect the extent to which the recruiters try to influence peers to access the intervention. This approach is premised on the idea that peers are better able to influence each other than an outsider. Having an IDU participant recruit helps to reach less visible IDUs in the population, who otherwise could not be reached by ORWs.

We undertook a longitudinal cohort study to examine HIV incidence and behavior change pre- and post-introduction of comprehensive HIV prevention services. We enrolled IDUs living in Delhi using multiple recruitment strategies. This paper presents information on the recruitment process and the outcome of the recruitment.

## Methods

Participants in this study consisted of men and women who injected drugs at least once in the last three months, were aged 18 years or older, resided in or around Delhi, were willing to participate in the study and provided written consent. The protocol was approved by the Institutional Review Boards of Population Council and PATH and the Ethics Committee of NACO.

### Recruitment methods

Participants were recruited through three strategies simultaneously: peer-referrals, targeted outreach by ORWs and as walk-in clients. IDUs had to come to one of five fixed drop-in centers (DIC) operated by Sahara. DICs were located near hotspots where large IDU populations resided in the central, east, north-east, and north-west districts of Delhi. Hotspots were places where groups of IDUs congregate.

### Peer-referral method

For peer-referral, we used the recruitment principal of the PDI strategy. We selected 11 initial “seed” participants to start the peer-referral recruitment process. The initial participants were selected based on their large network and diversity with regard to hotspots, sex, and other key demographic characteristics. Recruited IDUs were then asked to recruit others in their network of IDUs. Recruiters were given a recruitment coupon and asked to give one portion of the coupon to the recruit and retain the other portion to claim their reimbursement (secondary incentive) for successful recruitment. Recruits were asked to come to the study site with the portion of the coupon they received from the recruiter. Recruits were linked with recruitment coupons with unique ID numbers, which were printed on both stubs of the coupon so that the recruiter-recruit link could be established in the database. We limited the number of recruitment coupons given to 5 per person. The quota on the number of peers they can recruit helps to diversify the characteristics of the sample [12].

A dual incentive structure was put in place, typical of PDI, whereby participants received a primary reimbursement of Rs. 40 (USD 0.80) (regardless of recruitment method) for participation to cover the cost of travel and time and a secondary reimbursement of a meal ticket (non-redeemable for cash) for each peer they recruited into the study. The meal ticket could be used in designated eateries for food. The eateries then submitted the meal ticket at the study site to receive reimbursement of money. The ethics committee of NACO prohibited the use of monetary reimbursement for recruitment for fear of facilitating drug use. They allowed only the modest primary reimbursement amount.

Every participant who was registered irrespective of recruitment mode was eligible to receive five recruitment coupons and bring in IDUs from his/her network. The number of coupons distributed was tapered off towards the end of the recruitment phase to avoid IDUs coming to the site for registration after the recruitment ended.

### ORW referral method

ORWs recruited IDUs at hotspots identified prior to study initiation. Hotspots are the most efficient way to contact and interact with IDUs since IDUs frequent these places to support their drug habit. A mapping exercise was conducted by ORWs to identify the hotspots in the area and estimate the number of IDUs who could be contacted. This exercise was necessary since the existence of hotspots were dependent on the availability of drugs and peddlers and level of police surveillance and raids in the area. ORWs obtained information about the hotspots (i.e., peddlers, operational characteristics such as time of selling drugs, modus operand of sale, IDU volume) from discussion with other IDUs and NGOs engaged in TIs in the area. ORWs went to hotspots early in the morning as that is typically the time IDUs buy drugs. ORWs then drew a map of the area linking the routes that IDUs would have to commute in the area. Geographical boundaries for coverage by specific DICs were demarcated based on this information. The mapping exercise provided approximate numbers of IDUs that each DIC was expected to reach and helped to determine the number of ORWs needed to cover the hotspots around the DICs.

Hotspots were mostly located in and around places where IDUs bought and injected drugs, and included open areas such as parks, under flyovers or railway bridges. IDUs often remain close to these spots to find informal work for pay. Peddlers (drug dealers) who provide IDUs with the pharmaceuticals or other drugs typically reside or run medical shops around this area. Many street-based IDUs also live in and around the hotspots while home-based IDUs frequent this area to buy or inject drugs.

Most ORWs were former IDUs (74.5%). ORWs who had never used drugs were, however, very familiar with the IDU community and the neighborhoods. One advantage of using former IDUs as ORWs was that they knew many of the current IDUs by name and knew where to find them. They also had better acceptance by the IDU community as they were less likely to be judgmental and would keep injection and procurement networks confidential.

The ORWs visited assigned hotspots daily and were trained to provide information to the IDUs about the study, which included completing a behavioral survey and an HIV test and the opportunity to recruit their friends into the study. ORWs were restrained from providing any behavior change messages during this phase prior to intervention roll-out. IDUs, who did not have the peer-referral coupon and were willing to participate, were given an ORW referral coupon and escorted to the DIC by an ORW and considered to have been enrolled through targeted outreach. IDUs who had a peer-referral coupon with them and were interested in participating, were also escorted to the DIC by an ORW, however, these IDUs were considered to have been enrolled through peer referral. All ORWs carried a Sahara identity card and a copy of a letter from the District Commissioner of Police (DCP) giving support to the study and for safety reasons they visited hotspots in teams of two. This was crucial in an environment where criminal activities are widely prevalent and police raids on the IDU community common.

### Walk-in

Walk-in clients were those IDUs who learned about the project and related services and came in on their own (without recruitment coupon from peer or ORW).

#### ***Screening and registration***

The DIC was open for registration from 9 am to 4 pm. To accommodate working IDUs, DICs were open on Sundays and one DIC opened at 7:30 am in the last one month of the study. Every IDU reaching the DIC for registration was screened by an ORW using a brief screening questionnaire, which asked candidates about the last time they injected, types of drugs used, cost of drugs, combination of drugs used for injection, quantity of drugs used and place drugs were purchased to help verify active IDU status. Candidates were also asked to show injection track marks on their body to confirm that they were active IDUs. Eligible IDUs were registered into a live database, developed using window based software, which captured information on mode of recruitment, including linking recruits to their peer recruiters (if they came through peer referral) or ORW recruiter (if they came through ORW referral). A photo was taken and saved against the participant’s ID number. The system developed allowed for the ability to synchronize data collected to a central data warehouse in a secured data center that permitted centralized data or participant look-ups. Data synchronization took place when Internet connectivity was available. Mobile broadband USB modems were employed for the remote or mobile-clinics to provide connectivity to the centralized database. Data on the centralized data warehouse was backed up nightly and routine quality control checks or edits were performed by the site investigators. Any updates to the database at one site were instantly available at all other sites.

Given the mobile nature of IDUs, it was anticipated that many of the IDUs would try to register at multiple sites. To prevent this, the database checked all new participants against specific characteristics in the existing database (name, age, gender, religion, marital status, address, height, weight, and forearm length). If a match was found, the site staff checked against the photograph of the participant to verify that the IDU had not been previously registered at any of the five DICs. Once registered, participants were given an ID card, which included the ID number, and name and address of the DIC where the IDU registered. Additionally, site managers and ORWs regularly reviewed photographs of registered IDUs in the database in all five DICs to identify duplicate registrations as they had become familiar with the IDUs during the follow-up phase.

#### ***Behavioral interview and HIV testing***

After providing informed consent, trained interviewers conducted face-to-face interviews in Hindi, the local vernacular. The closed-ended questionnaire elicited information on demographic characteristics, injection and sexual behaviors, drug use history, HIV/AIDS knowledge, HIV testing history, Hepatitis B and C knowledge and testing history, alcohol use, and use of existing programs for IDUs. To understand the network size and relationship to the recruiter, IDUs were asked how many other IDUs they knew by name and knew how to contact them and who their recruiter was. HIV testing was conducted per NACO guidelines using rapid HIV tests described elsewhere [14]. Clients who refused to undergo the blood test or whose veins were too scarred for blood to be drawn underwent the behavioral survey only and were categorized as untested participants.

#### ***Statistical analysis***

Analyses were conducted using STATA version 10.0 (College Station, TX). Chi-square test was used to compare categorical variables and non-parametric Pearson’s Chi square test was used to compare medians. We compared characteristics and behaviors of IDUs recruited through the different recruitment methods. We also compared participants who successfully recruited peers and those who did not recruit any peers.

#### ***Cost analysis***

We determined the unit cost of recruiting and registering IDUs into the study for each of the three modes of recruitment.

The staff required to complete the registration of IDUs at the DIC, irrespective of the mode of recruitment, consisted of the site manager, monitoring and evaluation officer and two ORWs who managed the flow of IDUs and screened IDUs. The 20 staff managing the registration at the 5 DICs worked 8-hour days, 7 days per week during the recruitment phase (plus 4-hour days on Sundays for six of those weeks), totaling 29,600 hours. The salary paid to this staff over the recruitment period of six months and the costs incurred for training them were divided by the total number of IDUs registered in the study to obtain the unit cost for registering IDUs.

For the peer-referral method, the costs incurred for printing of peer recruitment coupons and the value of food coupons that recruiters of peer-referred participants received as incentive for bringing in IDUs from their network, were divided by the total number of IDUs recruited through peer-referral and this amount was added to the unit cost for registration to obtain the unit cost for this recruitment method.

For the ORW referral method, the salaries paid to ORWs and travel costs that they incurred were divided by the number of IDUs recruited through ORW referral to obtain the unit cost of recruitment which was added to the unit cost for registration. A total of 41 ORWs (excluding 2 ORWs per site dealing with screening and IDU management: already included in costs) worked on recruitment on average 8-hour days, 6 days per week over the 6-month recruitment period (plus 4-hour days on Sundays for six of those weeks), totaling 52,152 hours. ORWs who were involved in recruitment did not work regular fixed hours as outreach had to be conducted at times that were best for reaching IDUs, which typically were early mornings or evenings.

For walk-in clients the only cost incurred were registration costs. Staff time taken for interviewing clients, conducting blood test and HIV counseling and the Rs. 40 that participants received as monetary compensation for participation were not included in the cost analysis since the focus of this cost analysis is recruitment.

## Results

Between May and October 2011, 3,921 IDUs were recruited into the study. The 95 IDUs who had registered at multiple sites and 8 ineligible IDUs were removed from the study sample leaving a total of 3,818 IDUs (males: 3,792; females: 26). Of the 3,818 IDUs; 53.6% registered through ORW referral, 26.3% through peer referral, and 20.1% were walk-in clients. Despite all efforts, very few female IDUs could be identified; over half were recruited through ORW referral. Demographic characteristics of the IDUs recruited by recruitment method are given in Table 1. IDUs who were recruited through ORW referral were significantly older than those who were recruited through peer referral or walk-in (Median age: ORW: 30; Peer referral: 28; Walk-in: 28). Peer referred IDUs were significantly more likely to be non-Hindu (45.1% versus ORW: 37.8% and walk-in: 31.4%) and living with family or relatives (50.7% versus ORW: 40.1% and walk-in: 39.8%) compared to the other two modes of recruitment. There were no differences in sex and type of employment of IDUs by recruitment mode. IDUs recruited by ORWs had significantly smaller network size (median: 8) compared to IDUs recruited by peer referral and walk-ins (median: 10).

**Table 1:** Background characteristics of injection drug users by mode of recruitment in Delhi, India (2011)

**Variables**	**Total (n = 3818)**	**Outreach worker referral (n = 2048)**	**Peer referral (n = 1003)**	**Walk-ins (n = 767)**	**P-value**
**Gender**					
Female	26 (0.7)	15 (0.7)	5 (0.5)	6 (0.8)	0.516
Male	3782 (99.1)	2026 (98.9)	995 (99.2)	761 (99.2)	
Transgender	10 (0.3)	7 (0.4)	3 (0.3)	0	
**Age**					
≤30 years	2169 (56.8)	1095 (53.5)	606 (60.4)	468(61.0)	<0.001
31-50 years	1448 (37.9)	827 (40.4)	360 (35.9)	261(34.0)	
>50 years	201 (5.3)	126 (6.2)	37 (3.7)	38 (5.0)	
Median age in years (IQR)	30 (24, 38)	30 (24, 40)	28 (23, 36)	28 (23, 36)	<0.001^a^
**Educational status**^**b**^					
Illiterate	1873 (49.1)	1025 (50.1)	492 (49.1)	356 (46.5)	0.280
Class1-6	971 (25.5)	502 (24.6)	270 (26.9)	199 (26.0)	
Class 7+	969 (25.4)	518 (25.3)	241 (24.0)	210 (27.5)	
**Marital status**^**b**^					
Married/Cohabiting	1401 (36.8)	765 (37.4)	389 (38.8)	247 (32.3)	0.009
Never married	1970 (51.7)	1035 (50.7)	495 (49.3)	440 (57.5)	
Divorced/widowed	440 (11.5)	243 (11.9)	119 (11.9)	78 (10.2)	
**Religion**^**b**^					
Hindu	2344 (61.6)	1271 (62.2)	548 (54.9)	525 (68.6)	<0.001
Non Hindu	1462 (38.4)	772 (37.8)	450 (45.1)	240 (31.4)	
**Regional origin**^**b**^					
Delhi	884 (23.2)	465 (22.7)	216 (21.6)	203 (26.6)	0.016
Adjacent to Delhi	1775 (46.6)	946 (46.3)	464 (46.3)	365 (47.8)	
Others	1150 (30.2)	633 (31.0)	321 (32.1)	196 (25.6)	
**Accommodation**^**b**^					
Living with family/relatives	1631 (42.8)	819 (40.1)	508 (50.7)	304 (39.8)	
Living in rented/paying guest home	631 (16.6)	361 (17.6)	159 (15.8)	111 (14.6)	<0.001
Living in street/slum/public places	1549 (40.6)	865 (42.3)	336 (33.5)	348 (45.6)	
**Employment**^**b**^					
Salaried job	330 (8.7)	151 (7.4)	100 (10.0)	79 (10.3)	
Daily wage	2145 (56.3)	1156 (56.6)	558 (55.6)	431 (56.4)	0.111
Self Employed	1045 (27.4)	573 (28.0)	269 (26.8)	203 (26.6)	
Unemployed	291 (7.6)	164 (8.0)	76 (7.6)	51 (6.7)	
**Network size**^**b**^					
0 IDUs	74 (1.9)	36 (1.7)	25 (2.5)	13 (1.7)	0.038
1 -5 IDUs	1302 (34.2)	730 (35.7)	337 (33.6)	235 (30.8)	
6-20 IDUs	1844 (48.4)	974 (47.7)	498 (49.7)	372 (48.8)	
> 20 IDUs	590 (15.5)	304 (14.9)	143 (14.3)	143 (18.7)	
**Median network size**	10	8	10	10	0.038^a^

^a^Non-parametric Pearson’s Chi square test for medians.

^b^Sub-groups may not add up to totals due to missing data.

Table 2 describes the HIV status and testing history and injection behaviors by recruitment strategy. Although there was no significant difference in HIV testing history by recruitment method, there was a difference in self-reported previous HIV testing status. Walk-ins were significantly more likely to have tested positive previously (13.0%) compared to those recruited by ORWs (7.8%) and peers (10.5%). Additionally, walk-ins (23%) were more likely to have undiagnosed HIV infection; in other words, among those who had never tested or previously tested HIV negative or did not know previous result, walk-ins (23%) were significantly more likely to have tested positive in our study compared to those recruited by ORW (17.9%) and peers (16.4%). Walk-ins (26.1%) were also significantly more likely to have tested HIV positive compared to IDUs recruited by ORWs (19.9%) and peers (19.1%). Walk-in IDUs (62.2%) were also slightly more likely to have engaged in risky injection practices in the last one month than those referred by ORWs (57.0%) and peers (58.6%). Lastly, walk-ins (28.1%) were the least likely to have been recent injectors (one year or less) (peer referral: 38.6%; ORW: 34.3%) and were the most likely to have used the needle exchange in the last three months (walk-ins: 43.7% versus ORW: 32.6% and peers: 30.0%).

**Table 2:** HIV status and injection behaviors of injection drug users by mode of recruitment in Delhi, India (2011)

**Variables**	**Total**	**Outreach worker referral**	**Peer referral**	**Walk-ins**	**P-value**
	**(n = 3818)**	**(n = 2048)**	**(n = 1003)**	**(n = 767)**	
**Prior HIV testing**^**a**^					
No	2327 (61.1)	1276 (62.5)	604 (60.2)	447 (58.7)	0.135
Yes	1481 (38.9)	767 (37.5)	399 (39.8)	315 (41.3)	
Reported HIV status at prior HIV testing	(n = 1481)	(n = 767)	(n = 399)	(n = 315)	
Positive	143 (9.7)	60 (7.8)	42 (10.5)	41 (13.0)	
Negative	1035 (69.9)	543 (70.8)	285 (71.4)	207 (65.7)	0.023
Do not know	303(20.4)	164 (21.4)	72 (18.1)	67 (21.3)	
**Current HIV status**					
Positive	800 (20.9)	408 (19.9)	192 (19.1)	200 (26.1)	0.001
Negative	2,835 (74.3)	1,529 (74.7)	770 (76.8)	536 (69.9)	
Untested/indeterminate	183 (4.8)	111 (5.4)	41 (4.1)	31 (4.0)	
**Place where usually inject drugs**^**a**^					
Own home	350 (9.2)	192 (9.4)	86 (8.6)	72 (9.4)	0.057
Another IDU/peddler’s home	41 (1.1)	21 (1.0)	10 (1.0)	10 (1.3)	
Workplace	57 (1.5)	31 (1.5)	15 (1.5)	11 (1.4)	
Public place (street, parks, abandoned building, public toilets)	3361 (88.2)	1797 (88.1)	892 (88.9)	672 (87.9)	
**Time since initiating drug use**					
One year or less	163 (4.3)	74 (3.6)	44 (4.4)	45 (5.9)	
Two to five years	666 (17.5)	315 (15.4)	216 (21.6)	135 (17.8)	
Six to ten years	1134 (29.8)	590 (28.9)	318 (31.8)	226 (29.8)	
Eleven or more years	1840 (48.4)	1066 (52.1)	421 (42.2)	353 (46.5)	0.002
**Time since initiating injection drug use**					
One year or less	1295 (34.2)	699 (34.3)	384 (38.6)	212 (28.1)	
Two to five years	1556 (41.1)	807 (39.6)	393 (39.5)	356 (47.2)	<0.001
Six to ten years	662 (17.5)	367 (18.0)	156 (15.7)	139 (18.5)	
Eleven or more years	272 (7.2)	164 (8.1)	61 (6.2)	47 (6.2)	
**Number of Injections on the last day of injection**					
One	1,531 (40.2)	840 (41.1)	412 (41.1)	279 (36.5)	0.065
Two	1294 (33.9)	684 (33.5)	320 (31.9)	290 (37.9)	
Three or more	986 (25.9)	520 (25.4)	271 (27.0)	195 (25.5)	
**Risky injection practices in the last one month**^**b**^					
Never	1555 (41.5)	869 (43.0)	402 (41.4)	284 (37.8)	0.046
At least sometimes	2190 (58.5)	1153 (57.0)	569 (58.6)	468 (62.2)	
**Had taken needle/syringe from NSE program in last 3 months**					
Yes	1211 (34.05)	639 (32.6)	276 (30.0)	296 (43.7)	<0.001
No	2346 (65.9)	1320 (67.4)	645 (70.0)	381 (56.3)	
	(n = 3675)	(n = 1988)	(n = 961)	(n = 726)	
**Undiagnosed HIV infection**^**c**^	681 (18.5)	356 (17.9)	158 (16.4)	167 (23.0)	0.002

^a^Sub-groups may not add up to totals due to missing data.

^b^Risky injection behavior index includes the following practices in the past one month: (i) Using used needle or syringe; (ii) Back/ front loaded/split drugs; (iii) Shared vial/cooker/container/cotton/filter/water; (iv) Received pre-filled injection; or (v) Drew up drugs from common container.^c^Tested HIV positive in this study among those never tested or previously tested HIV negative or don’t know previous test result.

### Peer referral method

Of the 3,818 participants, 2,936 participants (76.9%) received all five coupons, 244 (6.4%) received two coupons, and 638 (16.7%) did not receive any coupons as we needed to end registration. Of those given 2 coupons, 92.2% recruited no peers, 5.3% recruited one peer, and 2.5% recruited two peers. Of those given five coupons, 84.7% recruited no peers, 7.4% recruited one peer, 3.0% recruited two peers, 1.7% recruited three peers, and 3.2% recruited four or five peers. The main reason mentioned by IDUs for not recruiting peers was loss of coupons (either by the recruiter or by the peer). Of the total 992 food coupons that could be claimed (i.e., the recruiter successfully recruited his/her peers), only 45.9% were claimed.

Table 3 shows the comparison of participants who successfully recruited at least one peer into the study and those who did not recruit any peers. No significant difference was observed in network size, frequency of injection or duration of drug injection between these two groups. However, those who successfully recruited at least one peer were significantly more likely to be in a stable living accommodation or living with family or relatives compared to those who did not recruit any peers (51.1% versus 42.7%; p = 0.046).

**Table 3:** Characteristics of peer recruiters by performance (2011)

	**Recruited no peers (n = 2949)**^**a**^	**Recruited at least one peer (n = 231)**^**a**^	**P-value**
**Median network size**	10	10	
**Time since initiating injection drug use**			
<1 year	981 (33.5)	85 (37.4)	
2-5 years	1215 (41.5)	89 (39.2)	0.474
6-10 years	519 (17.7)	34 (15.0)	
>11 years	212 (7.3)	19 (8.4)	
**Risky injection practices in the past one month**^**b**^			
Never	1192 (41.3)	83 (36.9)	0.198
At least sometimes	1696 (58.7)	142 (63.1)	
**Number of injections on last day injected**			
One	1141 (38.7)	90 (39.0)	0.987
Two	1001 (34.0)	79 (34.2)	
Three or more	806 (27.3)	62 (26.8)	
**Accommodation**			
Living with family/relatives	1257 (42.7)	118 (51.1)	0.046
Living in rented/paying guest home	475 (16.1)	32 (13.9)	
Living in street/slum/public places	1214 (41.2)	81 (35.0)	

^a^Among IDUs who were given peer referral coupons by study staff.

^b^Risky injection behavior index includes the following practices in the past one month: (i) Using used needle or syringe; (ii) Back/front loaded/split drugs; (iii) Shared vial/cooker/container/cotton/filter/water; (iv) Received pre-filled injection; or (v) Drew up drugs from common container.

### ORW referral method

A total of 56 hotspots were identified around the five DICs. The hotspots were categorized based on the number of IDUs found at the respective hotspots. There were 3 very large hotspots (300 or more IDUs), 5 large hotspots (approximately 100–200 IDUs), 24 medium (approximately 30–99 IDUs), 16 small (approximately 10–29 IDUs) and 9 very small hotspots (fewer than 10 IDUs). Large and very large hotspots were typically located near temples where devotees distributed free food and others were located near large drug selling spots. Medium hotspots were often near a public toilet or abandoned building. Small and very small hotspots were typically secluded spots in a residential area such as uninhabited houses, behind water tanks or bus station.

A total of 51 ORWs were hired to assist with participant recruitment. ORWs were mostly males (86%), their ages ranged from 21–50 years, 27% were illiterate and 57% had only primary level education, 59% were married, and 73% had previously worked with IDUs. There were 7 female ORWs, of whom three were present from the beginning to the end of the recruitment phase. Each DIC had at least one female ORW. Gender of the ORW did not show a higher probability of recruiting same sex IDUs.

Of the 2,048 IDUs who were recruited by ORWs (n = 51), 31.8% came from very large hotspots, 20.7% came from large hotspots, 36.9% came from medium hotspots, and 10.6% came from small or very small hotspots. ORWs recruited 55.9% (651/1165) of the IDUs found at very large hotspots, 55.1% (423/768) of the IDUs at large hotspots, 52.6% (757/1440) of the IDUs at medium hotspots, and 48.8% (217/445) of the IDUs at small or very small hotspots.

### Cost analysis

For the walk-in recruitment method, the cost per IDU recruited amounted to Rs. 375 (US $7.50), making the total cost incurred for recruitment through walk-in method Rs. 288,162 (US $5,763). The cost per IDU recruited through peer referral was Rs. 420 (US $8.40); the total cost incurred was Rs. 422,072 (US $8,441.40). The ORW referral method cost a total of Rs. 1,667,202 (US $33,344). The cost per IDU recruited through ORW referral method was Rs. 815 (US $16.30), making it the most resource-intensive method in terms of money, time and persons required.

## Discussion

Given the hidden nature of the IDU population, it is extremely challenging for service providers to identify and reach them for services. Even in Delhi, where a large proportion lives and injects in public, there is still a sizable proportion that is not visible and cannot be reached by traditional outreach methods. PDI approaches have been used to reach members of hidden populations who would otherwise not be reached by traditional outreach methods [10-13]. For this intervention study, we used peer referral along with ORW referral and walk-in method for recruiting IDUs. We ultimately found that for this population of IDUs in Delhi, ORWs were the most successful in recruiting IDUs for the study. However, having a mix of recruitment methods was advantageous in diversifying the sample enrolled into the intervention as there were differences in background and risk profiles of IDUs by recruitment method.

The relative success of ORW recruitment method over the other two recruitment strategies may be, in part, due to the level of training the ORWs received to motivate IDUs to participate in the study. ORWs were likely able to better motivate the IDUs and convince IDUs of the benefits of participation as opposed to peer recruiters who would not have the in-depth understanding of the study and the intervention as the ORWs. The majority of the ORWs in this study had prior experience of working with IDUs, and hence they not only knew the IDU community and hotspots very well, but they had already been sensitized to working with this population and knew the issues that may affect IDUs’ willingness to participate. Lastly, the IDUs that ORWs reach may be considered ‘low hanging fruits’ as they are easily identifiable and congregate openly in public places.

Although the ORW recruitment method was highly successful, it was also more resource intensive than the other two methods. The number of IDUs at a hotspot affected the cost of ORW recruitment since travel costs increased per IDU recruited from small or very small hotspots. On the other hand, in the case of larger hotspots, it proved to be more time-intensive as it was challenging to identify IDUs who had not yet registered in the intervention. The higher cost, however, should not prohibit programmers from using the ORW recruitment method, as outreach is an integral component of and highly effective strategy for harm reduction for IDUs [7-9]. Although in this study, ORWs were specifically instructed not to impart any harm reduction messages to IDUs as our goal was to have a pure baseline behavioral survey, future programs should use ORWs for both recruitment as well as harm reduction counseling.

The three recruitment methods yielded very different types of IDUs, which is useful in expanding coverage of programs. While ORWs were more likely to recruit street-based IDUs, the peer referral method was able to better access IDUs who were home-based and living with family or relatives. Further, we found that walk-in IDUs had higher risky injection behaviors. Walk-ins were also more likely to report previous HIV positive test, which may indicate their desire for services. Additionally, they were more likely to have undiagnosed HIV infection, suggesting a higher perceived risk and a desire for services.

The inclusion of peer referral method assisted in reaching many home-based IDUs who may not have been recruited if there was only ORW referral method [10,15]. The peer referral approach is more private and anonymous compared to being recruited by an ORW. Residential communities were reluctant to allow ORWs to visit their areas as persons interacting with them would be labeled as IDUs, thus limiting their access to home-based IDUs. Further, we learned that street-based IDUs were not as effective as home-based IDUs in recruiting their peers. Their unstable lifestyle and chaotic living environments (i.e., moving and working at different spots based on drug availability and injecting and sleeping in secluded spots such as near a drain or public toilet) likely make it challenging for them to keep track of their recruitment coupons; many IDUs mentioned that their recruitment coupons had got lost. Given that home-based IDUs were better at recruiting their peers compared to street-based IDUs, this presents an opportunity to take advantage of home-based IDUs to recruit their peers for interventions.

One of the challenges faced in this study with regard to peer referral recruitment was identifying an acceptable level of reimbursement for recruiting their peers. Despite presenting to the ethical committee of NACO of the need and ethical arguments (i.e., justice, beneficence, and respect) for using financial reimbursement for recruitment [16], the committee ultimately did not allow financial remuneration for recruitment fearing money would further encourage their drug use. Only the modest reimbursement of Rs. 40 (US $0.80) for travel and time irrespective of the mode of recruitment was allowed as financial reimbursement. However, as noted, less than half the recruiters actually claimed their food coupon, which suggests that this was not a sufficiently appealing compensation, which may have resulted in slow and inefficient recruitment. In a study we conducted in 2006–07 with IDUs in Delhi in which only respondent-driven sampling was used, money was given as compensation both for participation in the study as well as for successful recruitment. Unclaimed compensation by recruiters were minimal, and the study successfully recruited 800 IDUs from Delhi within a period of four months [3]. Thus, the provision of sufficiently appealing compensation is crucial to the success of using peer referral recruitment. Financial remuneration for both participation as well as recruitment has been successfully used in many studies that use RDS to recruit participants. Further, there are ethical arguments for providing such remunerations (i.e., respect for the participant’s time and effort) and safeguards can be placed so that remuneration can be provided in an ethically sound manner (such as placing quotas on number of recruits and keeping remuneration modest) [16].

Some limitations should be kept in mind. The study was not intended for comparing the feasibility and effectiveness of recruiting IDUs through different recruitment methods. So we cannot make conclusions about which recruitment method is more effective than the other methods. The study could only describe the process and the operational details of the three recruitment methods. Second, many IDUs indicated that although a peer had referred them to study, they no longer had the coupon with them. Because IDUs were counted as having been recruited by a peer only if they arrived at the study site with the peer recruitment coupon, some IDUs may have been misclassified as walk-ins and ORW-referred IDUs. Lastly, given the large number of IDUs recruited by ORWs, it is also possible that the IDUs who would have been accessible through the PDI strategy had already been approached or knew that they would eventually be approached by an ORW. Unfortunately, we do not have a record of refusals and acceptances of the ORW approach.

## Conclusion

In conclusion, this analysis of recruitment showed that a flexible approach of adopting different recruitment strategies simultaneously can, to a great extent, address the challenges faced in expanding coverage of targeted interventions to this high-risk and hard-to-reach population. The findings provide the basis to recommend a mix of recruitment methods in order to increase the diversity of IDUs enrolled in an intervention. The mixed method allows for IDUs with different demographic characteristics and risk profiles to be part of the intervention. Although the ORW referral approach was slightly more expensive than the other two methods, it was more successful in yielding the highest number of enrolled IDUs. It is crucial for current programs with IDUs to review their existing recruitment procedures and consider a mixed recruitment methodology used in this study.

## Competing interests

The authors declare that they have no competing interests.

## Authors’ contributions

WT conceived of the study and the design, and drafted the manuscript. MPS coordinated the data collection, conducted the analysis, and drafted the manuscript. VS oversaw the data collection, and participated in the conception of the manuscript. IM oversaw the data collection and contributed significantly to drafting the recruitment text of the manuscript. SS designed the live database and assisted with data collection. IT and DL contributed to the study conception and design. AS conceived of the study and the design, supervised the data collection, and provided substantial technical input in the conception and revision of the manuscript. All authors read and approved the final manuscript.
